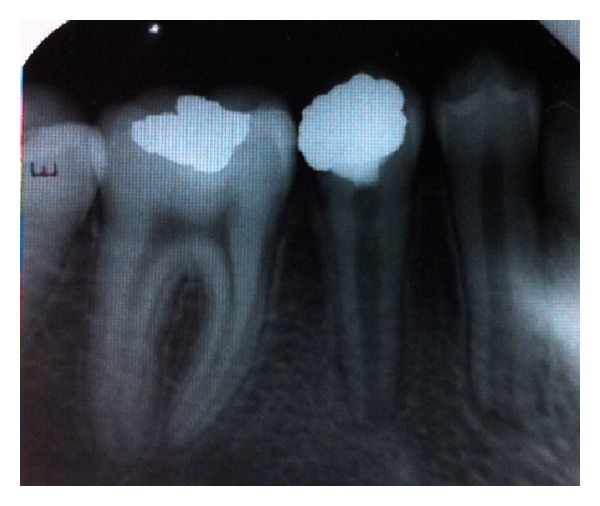# Induction of Maturogenesis by Partial Pulpotomy: 1 Year Follow-Up

**DOI:** 10.1155/2013/975834

**Published:** 2013-11-13

**Authors:** A. Bacaksiz, A. Alaçam

**Affiliations:** Department of Pediatric Dentistry, Gazi University, 06510 Ankara, Turkey

## Abstract

In cariously exposed immature permanent teeth, the treatment choice is controversial in pediatric dentistry. Radical root canal treatment usually appears to be the solution for these teeth. Even partial pulpotomy is a vital treatment for traumatically exposed immature permanent teeth; extending the borders of indication towards cariously exposed immature permanent teeth with reversible pulpitis may abolish the necessity of pulpectomy. This article describes the partial pulpotomy of a cariously affected immature permanent teeth and the follow-up for 1 year. A healthy 11-year-old male patient was referred to Gazi University Faculty of Dentistry Department of Pediatric Dentistry. The patient had reversible pulpitis symptoms on teeth numbered 45. At radiographic examination, immature apex and deep caries lesion were observed and partial pulpotomy was performed by using calcium hydroxide to maintain vitality of the pulp and allow continued development of root dentin expecting the root will attain full maturity. Clinical and radiographic follow-up demonstrated a vital pulp besides not only closure of the apex (apexogenesis), but also physiologic root development (maturogenesis) after 1 year. Partial pulpotomy is an optional treatment for cariously exposed immature permanent teeth for preserving vitality and physiological root development.

## 1. Introduction

The treatment of cariously exposed permanent teeth should maintain an arrestment of carious process and vital pulp free of inflammation [1]. The aim of vital pulp therapy is to protect the vitality and function of the coronal or remaining radicular pulp tissue [2]. If cariously exposed teeth not treated, pulpal necrosis or periradicular lesions may occur and patients may complain about pain, swelling, or any discomfort [3]. The treatment of option can be decided due to the maturation of the teeth. In deciduous teeth the aim is maintaining the function thereby, removing the infected coronal pulp or mummifying the radicular pulp tissue are the options. In permanent teeth, the border extends and usually following a pulpectomy, obturation of the root canal system is performed. 

Different factors such as trauma or caries can affect immature permanent teeth's root development and maturation. If the damage occurs including the pulp of the immature permanent teeth, the treatment becomes a challenge. It has been stated that pulpectomy is the best treatment choice to prevent and/or heal apical periodontitis [4]. It has been proved that, root canal treatment on vital teeth shows succeeding results [5, 6]. Nevertheless, the survival rate of endodontically treated teeth is not as successful as vital teeth, especially in molars (hazard ratio, 7 : 1) [7]. Because of that, preserving a vital pulp should be the main goal of treatment options. In cariously exposed immature permanent teeth, besides pulpectomy, vital pulp therapy is an alternative procedure because of the high healing capacity of pulp tissue in young patients [8–10]. By this procedure, while treating the reversible pulpal symptoms, the tissues remain vital and physiologic dentin deposition and root development continues. Thus, further interventional approaches such as root canal treatment may become unnecessary [11].

The overall success of vital pulp therapy mainly depends on which technique is performed, the inflammatory status of the teeth, the type of the agent which is used, the success criteria, and the period of follow-up [12].

 As Cvek described in 1978 [13], partial pulpotomy is a form of vital pulp therapy that consists of the surgical amputation of 2 to 3 mm of damaged and inflamed coronal pulp tissue. After removal of the damaged tissue, a dressing agent is placed to stimulate healing and maintain vitality of the remaining pulp [14]. It has showed successful results in the treatment of complicated crown fractures (95%) [15] and in cariously exposed immature posterior, asymptomatic permanent teeth (91–93%) [16, 17]. 

Calcium hydroxide, has been the material of choice to stimulate dentin formation after a carious exposure of immature permanent teeth for many years [11]. Cvek has reported that partial pulpotomy and pulp capping by using calcium hydroxide in immature permanent teeth had a success rate of 96% [13]. With calcium hydroxide application, a dentine bridge will consist above the healthy pulp tissue. In the absence of a dentin bridge, the remaining pulp becomes unattached which is followed by degeneration, atrophy and shrinkage from the dentine. Therefore, a dentin bridge formation provides a physical barrier from the external factors to protect the pulp. 

Not only calcium hydroxide but also mineral trioxide aggregate (MTA) has been suggested as a suitable material to be used in vital pulp therapy [18]. It has good physical characteristics and is biocompatible [19]. It also provides a good seal [20, 21] and has great marginal adaptation [22]. Moreover, it was shown in vitro that MTA did not induce apoptosis of pulp cells but instead induced proliferation of these cells [23]. 

The term “maturogenesis” has been used for the continued physiologic root development which is not restricted to the apical segment [24]. In this manner, the continued deposition of the root dentin provides a resistant structure against fracture. A case report is presented in which the vitality of the immature teeth maintained after performing an Ca(OH)_2_ partial pulpotomy allowing the physiological root development.

## 2. Case Report

**Figure 1:**
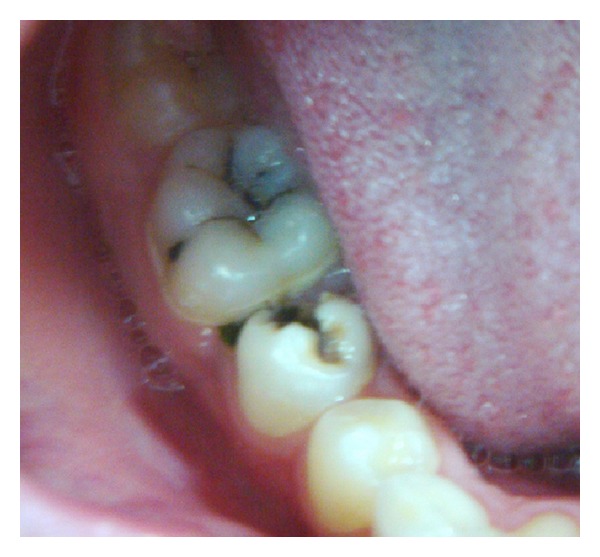


**Figure 2:**
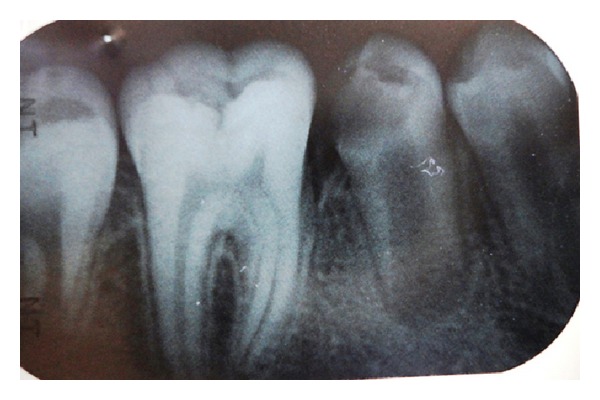


An 11-year-old Turkish boy consulted to the Gazi University Faculty of Dentistry Department of Pediatric dentistry clinic (Ankara, Turkey) with sensitivity to cold and sweets. No spontaneous pain was reported by the patient. The medical history was noncontributory. In the clinical examination gross occlusodistal caries was observed on his mandibular right second premolar (number 45) without signs of extraoral or intraoral swelling or sinus tract formation (Figure 1). The lower second premolar tested negative to percussion and palpation tests and the mobility was within the normal limits. Pulp vitality tests using electric pulp test showed a normal positive response without lingering sensation. Additionally, the adjacent teeth responded positive and within the normal limits to the electric pulp test. Radiographic examination revealed a close relation between caries and the pulp horns and undeveloped root with a wide-open apex and no evidence of periradicular pathology (Figure 2).

As a result of clinical and radiographic assessments, the pulpal status of the lower right second premolar was determined as vital with reversible pulpitis due to caries. The treatment plan included the removal of the carious lesion and clinical evaluation of the pulp exposure. Vital pulp therapy including partial pulpotomy with calcium hydroxide was planned and an informed consent was obtained from patient's father. 

**Figure 3:**
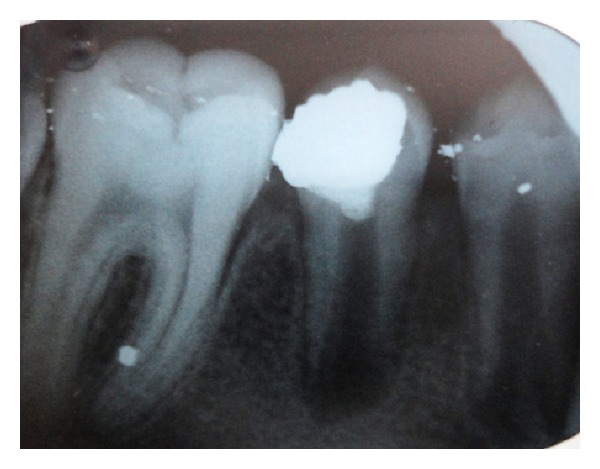


After local anesthesia, the tooth was cleaned with pumice and isolated with a rubber dam. Caries removal was performed with a sterile high-speed 801-016 ML diamond round bur with copious irrigation which was continued with steel round bur and exposure of pulp horns with moderate bleeding was observed. The amputation of the 2-3 mm damaged pulp was executed. The cavity was rinsed with saline and a sterile cotton pellet moistened with saline was used to apply moderate pressure to the exposed pulp for 5 min. After the homeostasis was achieved, calcium hydroxide was gently placed on the exposed pulp. A glass ionomer cement (Kavitan Pro, Spofa Dental, Czech Republic) was used as base material and the tooth was restored with amalgam (Figure 3). 

The patient was scheduled for 1 month follow-up in order to assess root developmentvitality and examine any signs or symptoms. The parents were informed to call the dental clinic if the patient reported any pain or discomfort. 

**Figure 4:**
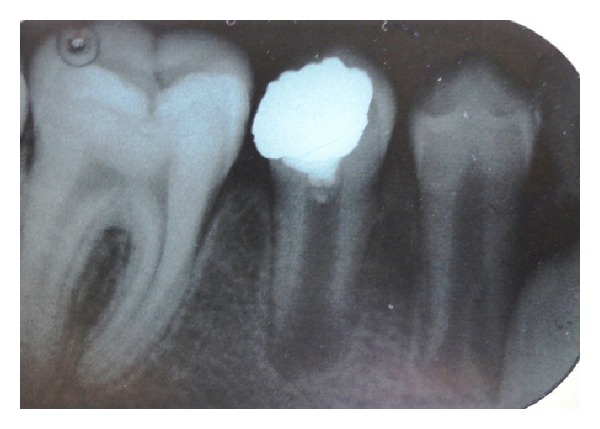


At the 1-month follow-up, the clinical examination showed an intact restoration and absence of any abnormal signs or symptoms. The patient reported no pain or discomfort. The tooth tested positive to the electrical pulp test and radiographic examination showed continued development of root and maturation of the root (Figure 4).

**Figure 5:**
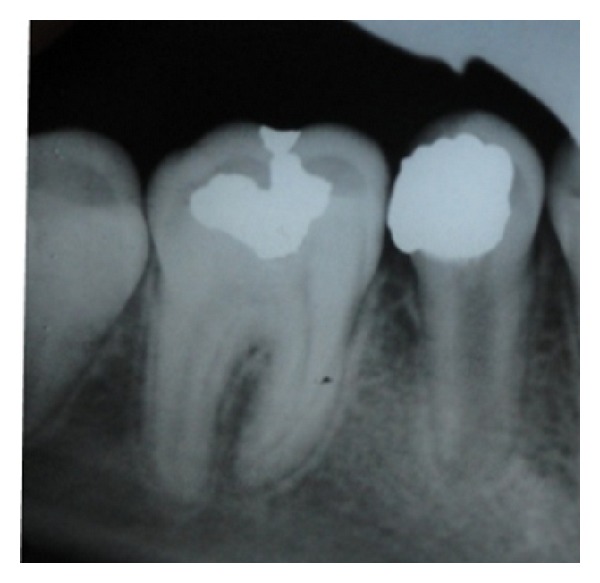


**Figure 6:**
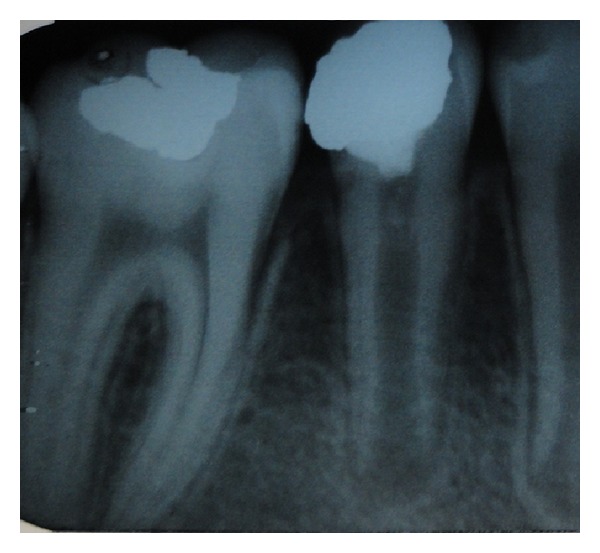


At 3 and 6 months, no abnormal findings were observed, and the clinical examination revealed no changes from the previous visit. The pulp continued to respond positive to the electrical pulp test. Root maturation was observed as a significant progression when compared with the preoperative radiographs (Figures 5 and 6). 

The patient was reexamined 12 months after treatment began, and reported as asymptomatic. The pulp tests repeated and the tooth responded normally to the tests. A periapical radiographical assessment showed continued root development and apical closure (Figure 7). Root maturation (maturogenesis) appeared normal with no evidence of internal root resorption or pulp calcification.

## 3. Discussion

Recently, vital pulp therapy and regeneration of infected pulps have become an alternative treatment procedure for immature permanent teeth [25, 26]. The aim of vital pulp therapy is to protect the reversibly damaged pulp from further injuries and promote the reformation while sustaining vitality [25]. Hence, as dentin deposition continues, a more resistant and mature root will produce against fractures [27]. Zilberman et al. [28] reported that 14 of 15 deeply carious immature permanent teeth showed a vital pulp response at 12–99 months following partial pulpotomy. Mass and Zilberman [17] found successful results up to 91.4% after a minimum of 12 months, performing partial pulpotomy in the treatment of cariously exposed immature permanent molars. 

While deciding the treatment options of an exposed teeth, the main consideration should be the degree of infection and inflammation in the pulp, instead of the size or duration of pulp exposure [8]. In contradiction to traumatic exposures, carious can lead to evident modifications in the pulp-dentine structure. These modifications are relevant to the degree of the disease and the maturity of the pulp [29]. Therefore, the important concern should be the selection of the case and treatment planning to acquire better results [30]. 

Although many new agents were introduced to the market, Ca(OH)_2 _ is still a well-accepted material to stimulate dentin formation after a carious exposure of immature permanent teeth [11]. In the literature, many studies reported that partial pulpotomy of cariously exposed immature permanent teeth with calcium hydroxide have sustained a success rate of 93 [16, 17, 28]. It is still the most accessible material and dentists can perform easily in clinics. Çalişkan [2] reported twenty-six permanent vital molars with carious pulp exposures and radiographic periapical involvement were treated using a full coronal pulpotomy, with calcium hydroxide placed directly on the radicular pulp tissue once bleeding had stopped. Of these, twenty-four teeth showed resolution of radiographic periapical involvement and dentine bridge formation after 16–72 months. The presence of radiographic periapical change accompanying reversible pulpitis can be as a sequel to reversible pulpal inflammation, rather than as a direct response to an infected pulp. 

Clinical and radiographical evaluations at 1, 3, 6, and 12 months showed pulpal vitality maintenance. Current vitality tests are still based on neurological inducement, and this is still a contradictory in immature teeth [12]. The radiographical assessment of periapical pathology in immature teeth is also questionable due to the normal radiolucency of the developing root.

In spite of the fact that histological success cannot be evaluated, clinical success can be assessed by the absence of any clinical or radiographic signs of failure and the substantiation of the development of the root dentin and apex closure. Thus, the patient should be recalled and reexamined periodically against any failure even if absence of visible symptoms. 

The optimal choice of treatment is, to maintain vitality of an exposed pulp rather than replacing it with a root filling material. However, the technique, the type of the agent which is used, the inflammatory status of the teeth is variable and a challenge to the dentists.

In the light of the literature and this case, for cariously exposed immature permanent teeth, partial pulpotomy is an optional treatment for preserving vitality and physiological root development. In this manner, as well as a vital pulp, not only closure of the apex (apexogenesis) but also physiologic root development (maturogenesis) occurs. 

## Figures and Tables

**Figure 7:**